# Label-free multiphoton microscopy as a tool to investigate alterations of cerebral aneurysms

**DOI:** 10.1038/s41598-020-69222-5

**Published:** 2020-07-23

**Authors:** Tina Sehm, Ortrud Uckermann, Roberta Galli, Matthias Meinhardt, Elke Rickelt, Dietmar Krex, Gabriele Schackert, Matthias Kirsch

**Affiliations:** 1 Neurosurgery, University Hospital Carl Gustav Carus, Technische Universität (TU) Dresden, Fetscherstraße 74, 01307 Dresden, Saxony Germany; 2 Neurosurgery, University Hospital Magdeburg, Otto-Von-Guericke University, Magdeburg, Saxony-Anhalt Germany; 3 Anesthesiology and Intensive Care Medicine, Clinical Sensoring and Monitoring, , Faculty of Medicine Carl Gustav Carus, TU Dresden, Dresden, Saxony Germany; 4 Pathology and Neuropathology, University Hospital Carl Gustav Carus, TU Dresden, Dresden, Saxony Germany; 5 CRTD/DFG-Center for Regenerative Therapies Dresden - Cluster of Excellence, Dresden, Saxony Germany; 6 National Center for Tumor Diseases (NCT), Dresden, Saxony Germany; 7 Asklepios Kliniken Schildautal, Seesen, Lower Saxony Germany

**Keywords:** Aneurysm, Cerebrovascular disorders, Multiphoton microscopy

## Abstract

Cerebral aneurysms are abnormal focal dilatations of arterial vessel walls with pathological vessel structure alterations. Sudden rupture can lead to a subarachnoid hemorrhage, which is associated with a high mortality. Therefore, the origin of cerebral aneurysms as well as the progression to the point of rupture needs to be further investigated. Label-free multimodal multiphoton microscopy (MPM) was performed on resected human aneurysm domes and integrated three modalities: coherent anti-Stokes Raman scattering, endogenous two-photon fluorescence and second harmonic generation. We showed that MPM is a completely label-free and real-time powerful tool to detect pathognomonic histopathological changes in aneurysms, e.g. thickening and thinning of vessel walls, intimal hyperplasia, intra-wall haemorrhage, calcification as well as atherosclerotic changes. In particular, the loss or fragmentation of elastin as well as fibromatous wall remodelling appeared very distinct. Remarkably, cholesterol and lipid deposits were clearly visible in the multiphoton images. MPM provides morphological and biochemical information that are crucial for understanding the mechanisms of aneurysm formation and progression.

## Introduction

Cerebral aneurysms represent local pathological dilatations in the vessel wall that predominantly appear near the bifurcations of the cerebral arterial circle^1^. Aneurysms can remain clinically silent until they rupture, which leads to a life-threatening subarachnoid haemorrhage associated with a high mortality and morbidity rate^2^.

A cerebral saccular aneurysm is an aneurysm verum characterized by bulging out of all three weakened vessel wall layers due to their high degree of pathological tissue alterations. Endothelial dysfunction of cerebral vessel walls leads to an inflammatory response, which triggers degenerative wall remodelling processes^3^ associated with multiple histopathological changes: a consistent tunica adventitia, sometimes with additional fibrinous material, a tunica media appearing thin or is even absent and an internal elastic lamina that is fragmented or often missing^1^. In addition, the normal endothelialized wall with linearly organized smooth muscle cells (SMCs) can undergo a thickening with a disorganization of SMCs; a hypocellularization of the vessel wall can occur as well as myointimal hyperplasia or luminal thrombosis^4^. Further histopathological alterations in cerebral aneurysm walls are associated with atherosclerotic changes such as lipid accumulation, e.g. deposition of cholesterol, presence of lipid-laden foam cells, oxidized lipids^5,6^ and calcification^7^.

Most of today’s knowledge about the mechanisms underlying aneurysm formation and disease progression was obtained by histopathological studies using conventional histological staining methods^4,5,8,6^. However, the origin of cerebral aneurysms, their initial formation as well as the progression to the point of rupture remain incompletely understood despite this wide range of research efforts. Therefore, additional imaging techniques on the microstructural level are needed to detect fine morphological and compositional changes that are crucial for understanding vessel wall remodelling, to uncover atherosclerotic changes and to provide the possibility to predict the risk of rupture.

Label-free multiphoton microscopy (MPM) including coherent anti-Stokes Raman scattering (CARS) microscopy in combination with endogenous two-photon fluorescence (TPEF) and second harmonic generation (SHG) could be helpful to fulfil this need. They visualize morphology and composition of different biological tissues and cells in a submicron resolution without photo-damage^9,10^. CARS imaging addresses molecular vibrations of CH_2_-groups in the tissue and, therefore, visualizes mainly the distribution of lipids^11,12^. This fact makes CARS microscopy a powerful tool for studying atherosclerosis^13^. TPEF microscopy exploits intrinsic cellular fluorescence originating from endogenous fluorophores like mitochondrial NADH and flavoproteins^14,15^. Moreover, two-photon excited autofluorescence of extracellular elastin is important for studying vessel wall remodelling^16,17^. SHG visualizes highly ordered tissue structures, which are non-centrosymmetric like type I collagen fibers^18,19^.

Raman spectroscopy is another analytical and non-destructive tool allowing the accurate identification of biochemical composition of different types of tissue^20,21^. This technique revealed that atherosclerotic plaques in peripheral arteries predominantly consist of cholesterol, cholesteryl ester, triacylglycerols, proteoglycans and crystalline calcium, typically in the form of calcium apatite^22–24^.

In this study, we applied label-free and non-destructive MPM to assess pathological changes in the morphochemistry of the vessel walls of human cerebral saccular aneurysm domes on the microstructural level. Moreover, Raman spectroscopy was used to obtain detailed biochemical information at selected positions of these alterations.

## Results

### Unaltered cerebral arteries

**Figure 1:**
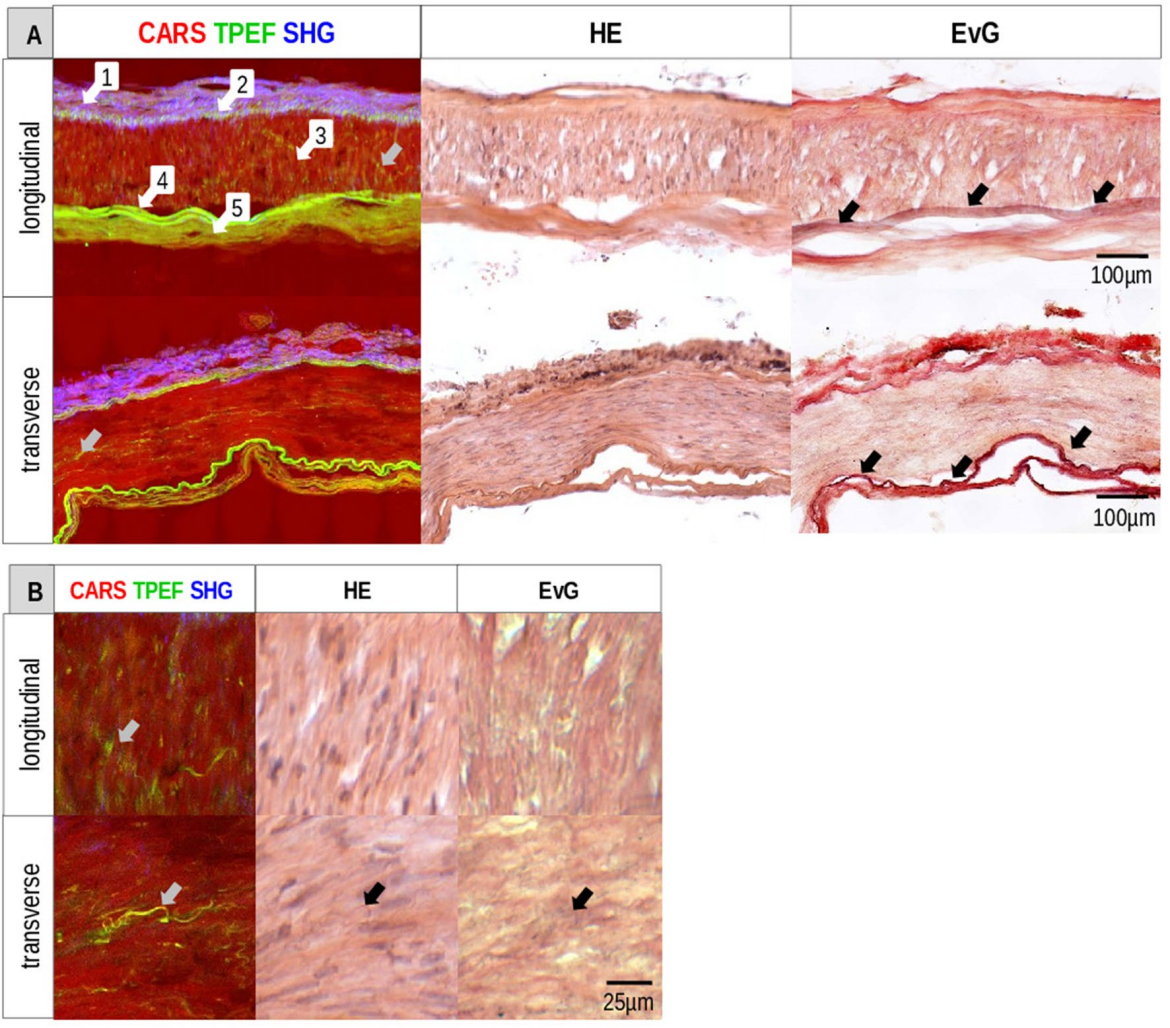
Normal vessel wall of the circle of Willis. (**A**) Longitudinal and transverse section visualized by MPM, stained with HE and EvG. 1-Tunica adventitia, 2-EEL, 3-Tunica media, 4-IEL, 5-Tunica intima. Grey arrows in the longitudinal and transverse section mark the elastic fibers in the tunica media. Black arrows indicate the IEL. (**B**) Higher magnification of the small elastic fibers from (**A**) marked by grey arrows.

MPM was conducted to investigate transverse and longitudinal sections of a regular vessel wall of human cerebral arterial circle. Conventional histopathological stainings for hematoxylin & eosin (HE) and Elastica van Gieson (EvG) were used as reference (Fig. 1A). EvG can visualize elastin-bearing tissue structures in black-purple and collagen-bearing structures in red. A normal cerebral artery consists of three layers: tunica adventitia (1), tunica media (3) and tunica intima (5). TPEF (colored in green) visualized the external elastic lamina (EEL) (2) and the internal elastic lamina (IEL) (4) very clear. SHG (colored in blue) showed the collagenous connective tissue of the tunica adventitia, consisting mostly of collagen type I. CARS (colored in red) displayed the tunica media, which is characterized by a linearly organized smooth muscle cell (SMC) layer. TPEF and CARS mainly visualized the tunica intima with its subendothelial stratum and the single layer of endothelial cells. Noteworthy, all structures of the normal cerebral vessel wall were identified with label-free MPM. Moreover, tissue structures appeared clearer with higher contrast compared to standard histological stainings. In particular, multiphoton images clearly revealed all elastic fibers, including the very thin and small ones within the tunica media (Fig. 1B). MPM imaging identified easily the abluminal-luminal orientation based on the strong SHG signal of the collagen-rich tunica adventitia, which was very helpful for the analysis. The tunica adventitia is always located at the top in the displayed images.

### Aneurysms

Label-free MPM was applied to study five unruptured (diameter 9–16 mm) and five ruptured (diameter 5–20 mm) human cerebral saccular aneurysm domes (Table 1). All the pathological tissue changes visualized by MPM in this set of experiments are in accordance with previous studies made in rabbits^25^, pigs^13^ and humans^26^. Therefore, we abstained from further special immunohistochemical stainings as reference. Instead, we used Raman spectroscopy to confirm the biochemical composition of different types of tissue visualized by MPM.

**Table 1:** Summary of the comparison of MPM with histopathological stainings regarding pathological vessel wall alterations during the process of aneurysm formation.

		Unruptured					Ruptured				
Patient		1	2	3	4	5	1	2	3	4	5
Size (mm)		9 × 11 × 10	9	14 × 11 × 11	9	15 × 16 × 16	5	6	10 × 6	20	> 10
OP-Age (years)		66	38	36	54	57	64	74	65	63	69
Location		MCA R-Bi	MCA R-Bi	MCA L-Bi	MCA R-Tri	PCoA R	ICA L-Bi	PCoA R	PCoA L	MCA L	PCoA L
Multiple Aneurysms		No	No	Yes	Yes	Yes	Yes	Yes	No	No	No
IEL Fragmented	MPM	–	–	√	–	(√)	–	–	–	–	–
	HE	–	–	–	–	–	–	–	–	–	–
	EvG	–	–	–	–	–	–	–	–	–	–
EEL Fragmented	MPM	(√)	(√)	√	(√)	(√)	–	–	–	(√)	(√)
	HE	–	–	–	–	–	–	–	–	–	–
	EvG	–	–	–	–	–	–	–	–	–	–
Elastin	MPM	√	√	√	√	√	–	√	–	√	√
	HE	–	–	–	–	–	–	–	–	–	–
	EvG	√	√	(√)	√	(√)	(√)	(√)	––	(√)	(√)
Lipid	MPM	–	–	–	√	√	–	–	√	√	√
	HE	–	–	–	–	–	–	–	–	–	–
Cholesterol	MPM	√	–	–	√	√	–	√	–	√	√
	HE	–	–	–	–	–	–	–	–	–	–
Calcification	MPM	–	–	–	√	–	–	–	–	–	–
	HE	–	–	–	√	–	–	–	–	–	–
Fibrotic Alterations	MPM	√	√	√	(√)	√	–	–	–	√	√
	HE	–	–	–	–	–	–	–	–	–	–
Intra-wall Heamorrhage	MPM	(√)	√	–	(√)	–	√	√	√	√	√
	HE	(√)	√	–	–	–	√	√	√	√	√
Disorganised SMC	MPM	√	√	√	√	√	√	√	√	(√)	√
	HE	√	√	√	√	√	√	√	√	(√)	√
Hypocellular Areas	MPM	–	–	–	–	–	–	–	–	–	–
	HE	–	–	–	√	√	–	–	√	–	√
Thinned Areas	MPM	–	–	–	–	–	–	–	–	–	√
	HE	–	–	–	–	–	–	–	–	–	√
Intimal Hyperplasia	MPM	√	√	√	(√)	√	√	–	–	√	√
	HE	√	√	√	(√)	√	√	–	–	√	√
Foam Cells	MPM	–	–	–	–	√	–	–	–	–	√
	HE	–	–	–	–	√	–	–	–	(√)	√

√—visible (√)—hardly visible – —not visible.

**Figure 2:**
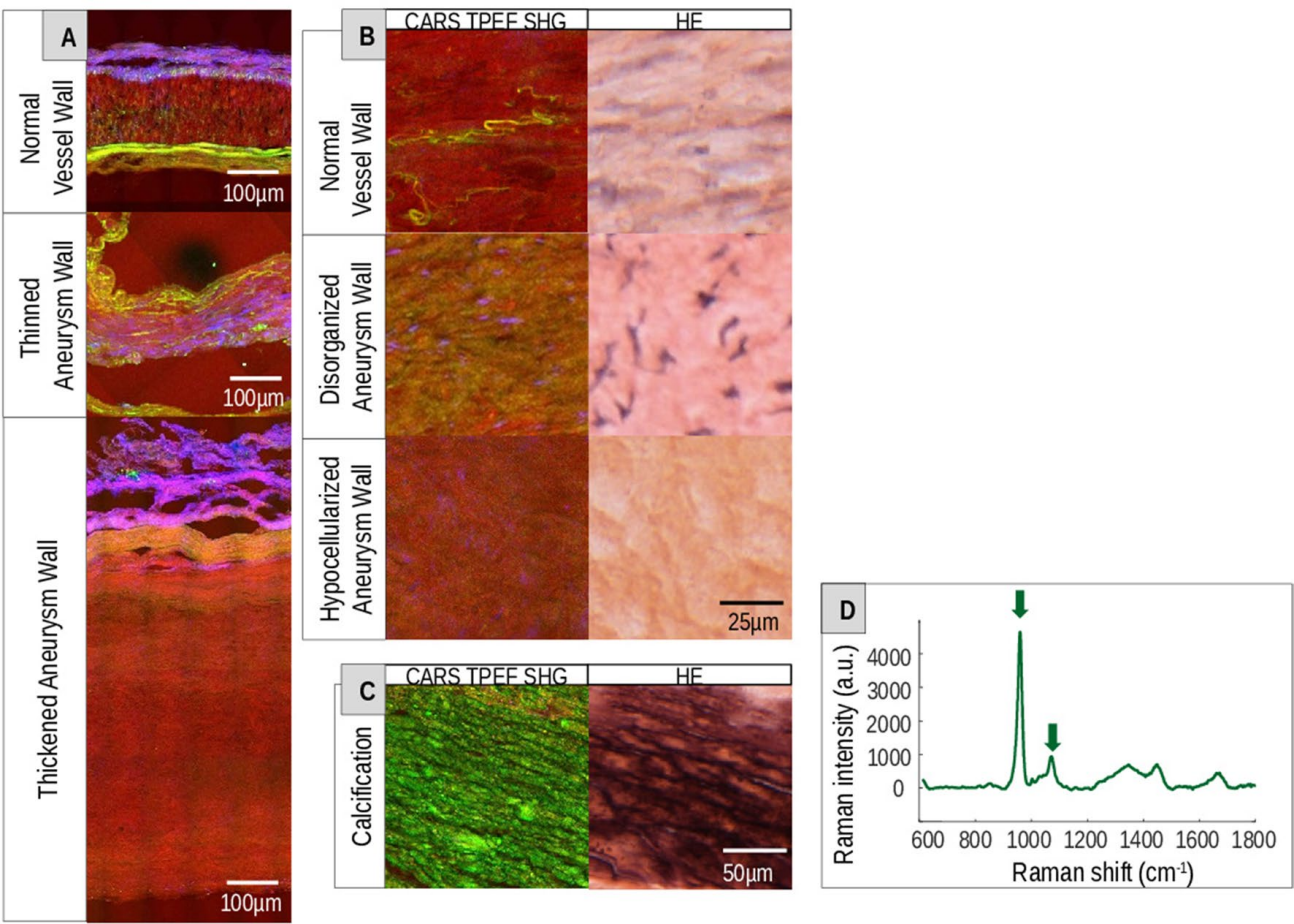
Pathological tissue alterations of intracranial vessel walls. (**A**) Multiphoton images of a normal vessel wall and thinned as well as thickened aneurysm walls. (**B**) Normal vessel wall and disorganized and hypocellularized aneurysm wall visualized by MPM in comparison to HE. (**C**) Multiphoton and HE images of a calcification in an aneurysm wall. (**D**) Raman spectra of the calcified area in (**G**). The arrows indicate the calcium hydroxyapatite (690 cm^-1^) and calcium carbonate apatite (1073 cm^-1^) bands.

Initially, pathological tissue alterations that were likewise visualized by HE staining and MPM were assessed (Fig. 2). Multiphoton images displayed areas with thickened and thinned aneurysm walls compared to normal cerebral vessel walls (Fig. 2A). Pathological intima thickening (pathological intimal hyperplasia) was found in almost all resected human aneurysm domes in contrast to normal vessel walls (Fig. 2A, Table 1). However, areas with disorganization as well as hypocellularized areas were better assessable in HE staining due to their distinctly stained nuclei (Fig. 2B, Table 1). Nevertheless, the multiphoton images showed a noticeable disorganization of the tissue structure in comparison to the normal vessel wall. Another histopathological alteration associated with vessel wall changes in aneurysms is calcification. In the multiphoton images, areas with high TPEF (green) signal matched regions of calcification that are visible in HE staining (Fig. 2C, Table 1). Raman spectroscopy was used to confirm the nature of the areas with high TPEF intensity. The spectra of those areas exhibited bands of calcium hydroxyapatite (690 cm^-1^) and calcium carbonate apatite (1073 cm^−1^)^27^ consistent with the presence of calcification (Fig. 2D).

**Figure 3:**
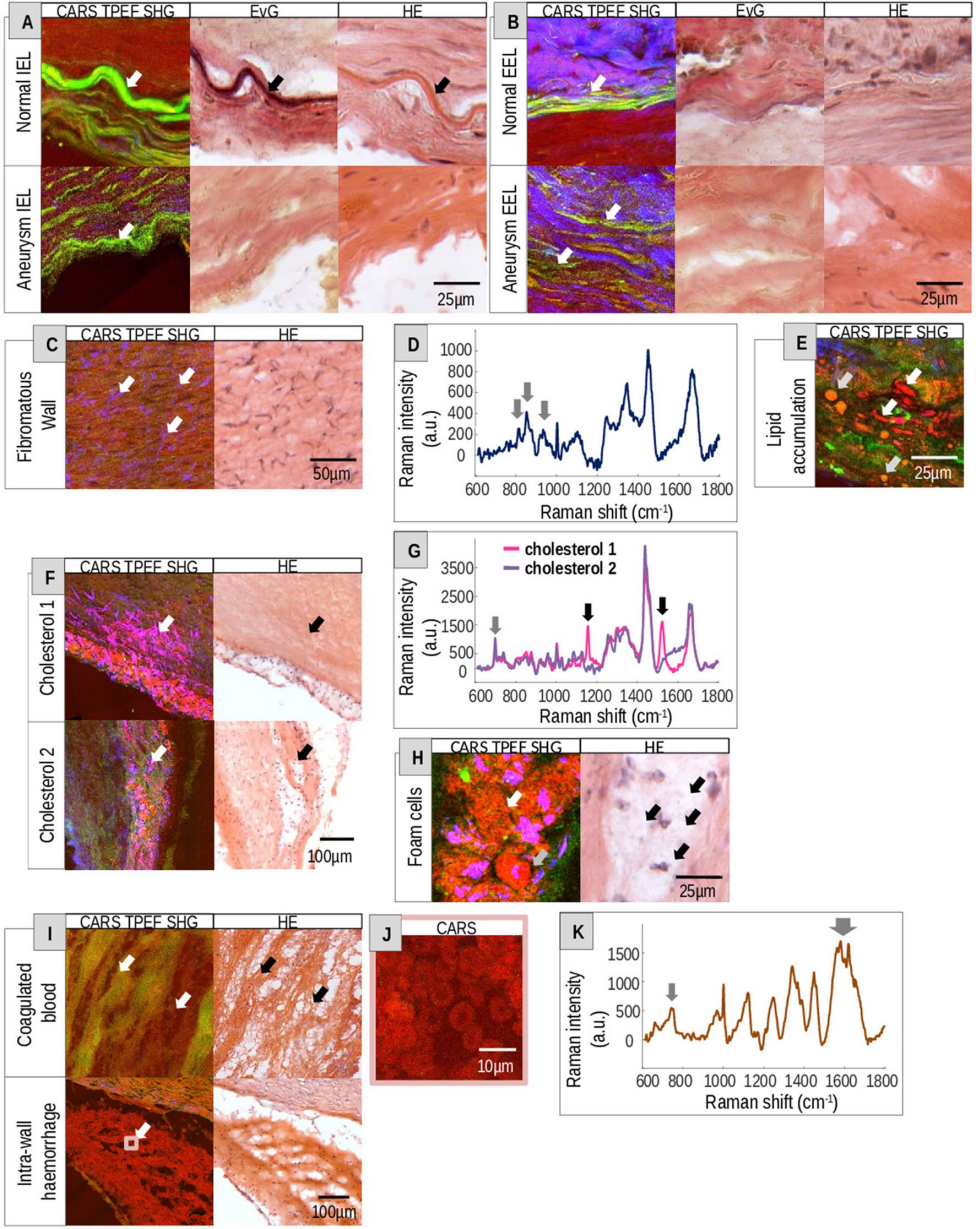
Pathological tissue alterations of intracranial vessel walls. (**A**, **B**) Label-free multiphoton images of the (**A**) fragmented IEL and (**B**) fragmented EEL in comparison to EvG and HE of normal vessel walls and aneurysm walls. Arrows indicate the elastic lamina. (**C**) Collagen accumulation (arrows) in an aneurysm wall. (**D**) Raman spectra acquired in the area of the fibromatous wall remodeling shown in **C**. Arrows indicate collagen bands (817, 855 and 933 cm^−1^). (**E**) Intra- and extracellular lipid accumulation (arrows) in an aneurysm wall. (**F**) Cholesterol deposits in an aneurysm wall (white and black arrows). The overlay of the SHG (blue) and CARS (red) signals visualize cholesterol crystals (magenta). (**G**) Raman spectra of cholesterol 1 and 2 (cholesterol 1, cholesterol 2) acquired in the region indicated in F. The gray arrow marks the cholesterol band (702 cm^−1^) and the black arrows the carotene bands (1158, 1521 cm^−1^). (**H**) Multiphoton and HE images of foam cells (black arrows) and lipids. Intra (white arrow) and extracellular (gray arrow) lipids are indicated. (**I**) Coagulated blood (arrows- indicate fibrin structure) and intra-wall haemorrhage shown by MPM and HE. (**J**) Red blood cells (RBCs-erythrocytes) of the intra-wall haemorrhage/thrombus from I. (**K**) Raman spectra of coagulated blood shown in I. Gray arrows indicate the bands related to RBC and heme groups of hemoglobin (752, 1563, 1575, 1620 cm^-1^).

As a next step, we found that some pathological tissue alterations related to aneurysm formation were visualized better by MPM than by conventional HE staining (Fig. 3).

Initially, the presence of the internal (IEL) and external elastic lamina (EEL) were assessed by MPM, HE as well as EvG (Fig. 3A,B). The elastic fibers were characterized by strong TPEF (green) signal and were better visible in the multiphoton images than in the EvG reference staining. In normal vessel walls, the sensitivity of EvG was sufficient to visualize the IEL and EEL. In addition, the IEL was visible in HE images as well. However, in aneurysm walls, the conventional stainings faced their limitations. The elastic laminas were not detectable by conventional histological stainings. In multiphoton images of aneurysms, the IEL as well as the EEL was fragmented or not visible (Table 1). Conspicuously, the EEL in aneurysms was no longer visible in its original compact and lined form (Fig. 3B). Furthermore, MPM was able to show the fibromatous remodelling in aneurysm walls by visualization of collagen accumulation by SHG (blue) (Fig. 3C, Table 1). Raman measurements validated the fibromatous changes by detecting the main bands associated with collagen at 817, 855 and 933 cm^−1^ ^27^ (Fig. 3D).

Atherosclerotic changes are associated with lipid and cholesterol deposits and their accumulation. MPM was able to visualize intra- and extracellular lipid deposits in atherosclerotic changes of aneurysm vessel walls (Fig. 3E,H). Small amounts of lipids were detected by the high intensity of the CARS (red) signal indicating the presence of C–H bond of lipids (Fig. 3E). Lipid-laden foam cells typically found in atherosclerotic changes were evident in HE images (Fig. 3H, black arrows). In the MPM images, clearly demarcated small lipid spots, which occurred in larger accumulations, might represent foam cells (Fig. 3H, white arrow). The large areas with intense CARS signal might represent extracellular accumulated lipids (Fig. 3H, gray arrow). In addition, deposits of cholesterol in aneurysm walls were clearly visible in MPM (Fig. 3F). Cholesterol crystals are characterized by intense CARS (red) and SHG (blue) signal, therefore, they appear as magenta structures in the MPM images^28^. HE staining did not show crystalline structured clefts of cholesterol (Fig. 3F). The decellularized wall structure (black arrow) of the cholesterol image 1 was characterized by clearly visible cholesterol crystals in the MPM image in contrast to the HE image (Fig. 3F). Raman spectra of cholesterol image 1 and 2 displayed the main cholesterol band at 702 cm^-1^ as well as other cholesterol ester bands at 429, 1087, 1130, 1299, 1301, 1442, 1464 and 1739 cm^−1^ ^27^ (Fig. 3G). Notably, Raman spectroscopy of the cholesterol 1 image also showed bands representing carotenoids at 1158 and 1521 cm^−1^ ^27^.

Furthermore, MPM and HE staining are able to visualize coagulated blood originating from a rupture and intra-wall haemorrhage in an aneurysm wall (Fig. 3I). Coagulated blood is characterized by its unique structure of fibrin from plasmatic blood clotting in HE images. In multiphoton images, the coagulated blood displayed diffuse TPEF signal. Additionally, MPM is able to directly visualize single red blood cells (RBCs) in the intra-wall haemorrhage that are recognized based on their typical morphology in the CARS image. Their round biconcave disc-shaped character with central pallor was visible at higher magnification (Fig. 3J). Raman spectra of those areas confirmed the presence of blood. Raman bands of hemoglobin at 752, 1563, 1575 and 1620 cm^-1^ ^27^ were detected (Fig. 3K).

Subsequently, an overview was generated summarizing the results of the comparison of MPM with the common histopathological stainings for H&E and EvG according to important histopathological alterations associated with pathological changes within the vessel wall during the process of aneurysm formation (Table 1). Most alterations were visible in both types of aneurysms, unruptured and ruptured; differences emerged more in the frequency of the occurrence. The differences we saw (e.g. fragmented IEL, fibrotic alterations and calcification) arise most likely from the small number of aneurysm domes we studied. However, the loss of elastin and collagen fibers in ruptured aneurysms are in line with the loss of elasticity and repair mechanism of the vessel wall during disease progression.

### Bifurcation

**Figure 4:**
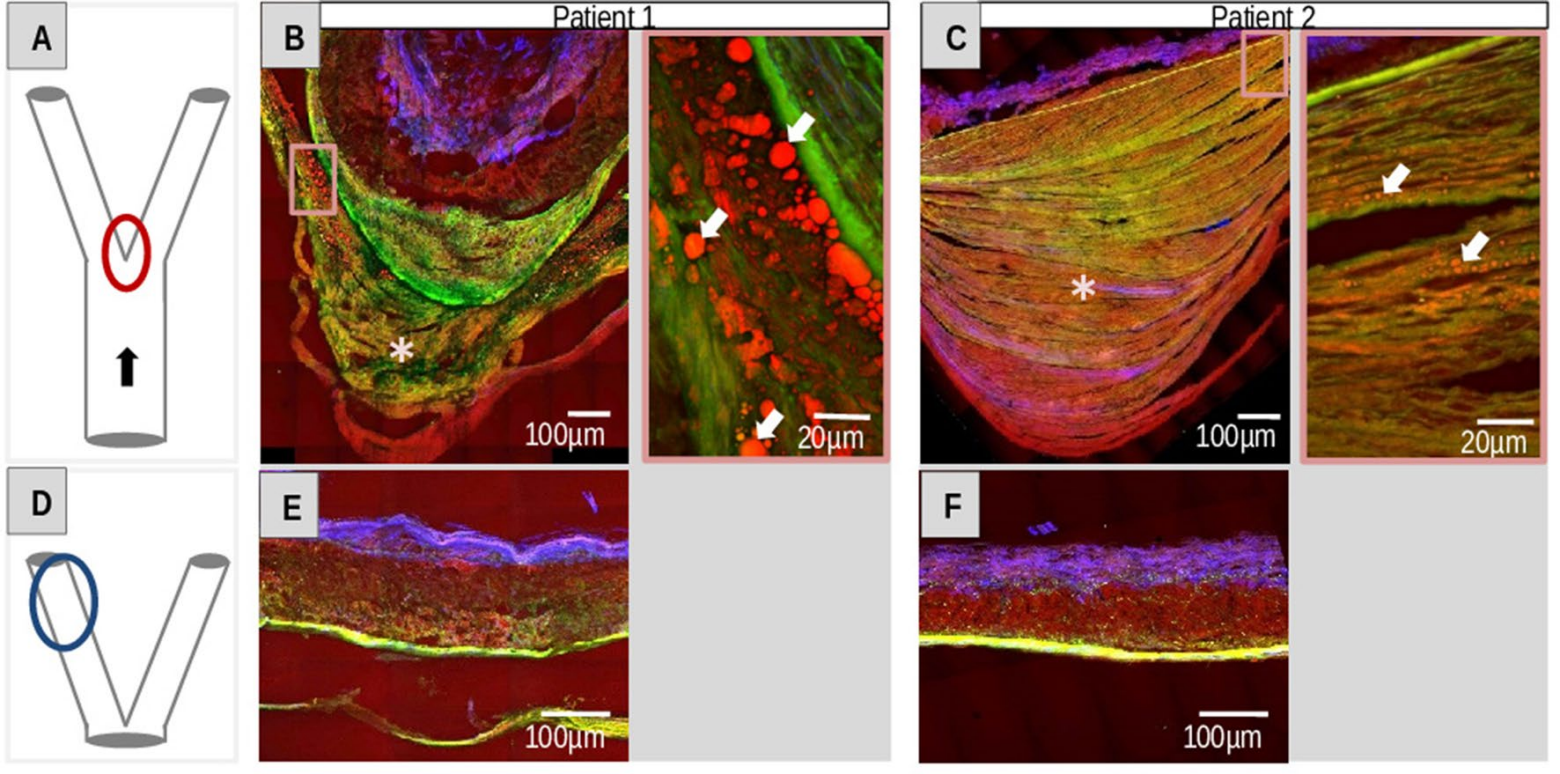
MPM images of intracranial vessel walls of bifurcations and distant from bifurcations. (**A**) Illustration of a bifurcation. The direction of blood flow (arrow) and the apex (red oval) region of high shear stress are indicated. (**B, C**) Vessel wall of bifurcations of cerebral arterial circle as visualized by MPM at different magnifications. White arrows mark lipid deposits in the thickened tunica intimas that in turn is marked by asterisk. (**D**) Illustration of the location (blue oval) of (**E, F**) vessel walls distant from bifurcations visualized by MPM.

Furthermore, MPM was used to investigate cryosections of bifurcations from the human cerebral arterial circle. The special hemodynamic situation of high shear stress on the branching points of vessel trees (apex-region of e.g. bifurcations) makes them susceptible to wall remodelling and atherosclerotic changes (Fig. 4A). This in turn also fosters the development of saccular aneurysms. Both bifurcations examined here displayed alterations of the vessel wall, and in particular an evident thickening of the tunica intima marked by asterisk (Fig. 4B,C). Patient 1 exhibited an intact muscular layer of the tunica media, the IEL was fragmented as well as thickened in a few areas and lipid accumulation (white arrows) was found near the IEL (Fig. 4B). Patient 2 showed a fragmented and thinned tunica media (Fig. 4C). However, the IEL was still intact but not organized as lamellae anymore. The lipid deposits (white arrows) were located close to the IEL and collagen deposits were visible in the thickened intima (Fig. 4C). In addition, we investigated by MPM the cerebral vessel wall structure distant from the two studied bifurcations (Fig. 4D) in order to verify whether the vessel wall alterations found in bifurcations are bifurcation-specific or patient-specific (Fig. 4E,F). Both vessel walls distant of bifurcations showed rather normal vessel wall structure. This observations support the idea that the alterations found in the vessel walls of the bifurcations were bifurcation-specific.

## Discussion

In this study, we highlight the application of MPM as label-free and non-destructive tool to assess the pathological remodelling processes of cerebral aneurysm vessel walls, including atherosclerotic changes, on the microstructural level with high resolution. In particular, alterations in presence, orientation and localization of elastin were easily recognized based on TPEF signal. Moreover, collagen of the adventita and of fibrous regions was visualized by SHG. CARS showed lipid droplets and lipid-laden cells and, if colocalized with SHG, indicated the presence of cholesterol. In addition, the crystalline structure of calcifications was visualized by TPEF and erythrocytes were detected by CARS.

Label-free MPM was already reported to represent a very beneficial, fast and real-time approach for the investigation of pathological vessel wall changes in peripheral arteries with the aim to study aging, fluid–solid interaction and cardiovascular diseases^13,25,26,29,30^.

MPM was used to investigate vessel wall stiffening of unruptured human cerebral aneurysm domes^30^ and in the ApoE mouse model of aneurysm^29^. Here, the combination of TPEF and SHG was applied to analyze structural reorganization of the extracellular matrix (ECM) microstructure. In particular, collagen and elastin fiber orientation and the degree of fiber alignment were addressed, which play an important role during onset and progression of arterial pathologies such as vessel wall stiffening^29,30^. In line with these previous findings, our data confirmed that MPM allows the visualization of fibromatous wall remodelling in cerebral aneurysms. The specific Raman bands of collagen supported the MPM findings of collagen accumulation. The results obtained by MPM on presence, fragmentation or full loss of the elastic laminas in aneurysm walls were consistent with the results of conventional EvG staining as shown by other groups^31^. Notably, MPM (TPEF) is able to visualize elastic fibers in cryosections more clearly.

Furthermore, MPM already assessed atherosclerotic plaque burden in different animal models such as pigs^13^ and myocardial infarction-prone rabbits (WHHLMI)^25^ as well as in human aortas^26^. In accordance with these studies, we showed that MPM is able to detect lipid-laden foam cells and lipid droplets in human cerebral aneurysm domes. The lipid accumulations in cerebral aneurysms are more diffuse compared to the more compact atherosclerotic plaque in peripheral arteries^5,6,8^. Apart from the lipid accumulation, low-density lipoproteins such as cholesterol esters are key components of an atherosclerotic plaque in coronary artery samples^32,33^. Multiphoton images showed cholesterol crystals within the vessel wall but those were not detectable in HE image.

Noteworthy, Raman spectroscopy demonstrated presence of carotenoids in atherosclerotic-altered areas in the studied cerebral aneurysms, which are absent in regular healthy tissues^34^. Carotenoids have already been found ex vivo in necrotic core regions of coronary arteries sections^33^. Moreover, the presence of carotenoids was confirmed also in vivo during carotid endarterectomy and femoral artery bypass surgeries by catheter-based Raman spectroscopy^35^, and in patients suffering from abdominal aneurysms^36^. This provides further evidence for the advanced pathological alterations of the studied cerebral aneurysms.

Another histopathological alteration occurring in cerebral aneurysms is calcification. MPM detected calcifications in human aortas by an intense TPEF signal of the crystalline structure of calcium^26^. Our findings show that MPM visualizes calcification in cerebral aneurysms as well.

Aneurysms are also associated with bleedings. Recently, it was shown that MPM is able to visualize single mature erythrocytes based on TPEF signal^37^. Our results confirm that MPM allows displaying the typical morphology of erythrocytes. However, we visualized erythrocytes with CARS signal and not with TPEF. The reason might be related to different excitation and signal collection schemes.

In addition, we investigated cerebral arterial bifurcations without saccular aneurysm with MPM. In particular, shear stress and pressure of the blood flow stream against the walls of bifurcations mechanically contribute to alteration of their vessel walls during normal aging. This can also foster the development of saccular aneurysms in peripheral arteries as well as cerebral arteries^38–41^. Histomorphological alterations during normal aging in cerebral arteries are a thickened intima and fragmented/flattened IEL^42,43^ as well as thickened tunica media with collagen deposits compared to young vessels with a thinner media^39^. In this study, one bifurcation showed in the MPM a normal tunica media without collagen deposits images only the thickened intima and the fragmented IEL were visible, which goes in line with the literature. The other bifurcation displayed an intact but flattened IEL in the MPM images, the tunica media was fragmented at the apex and the strong thickened intima showed collagen deposits. Sheffield and Weller reported this flattening of the IEL over a tunica media gap at the apex of patients above 60 years of age^42^. They also mentioned the fibrotic accumulations in the thickened intima of elderly people, which we also observed in the MPM images. Furthermore, they described lipid deposits in deeper layers of the thickened intima^42^. This confirms the hypothesis that lipid droplets accumulating in parts of the thickened intima in visible both bifurcations might be related to aging. In addition, we imaged vessels walls distant of the bifurcations that displayed a comparatively normal vessel wall structure. These findings support the conclusion that the alterations found in the bifurcation vessel walls were bifurcation-specific and not patient-specific.

Our findings provide a starting point for future MPM based investigations addressing the transition of normal vessel aging towards pathological events: This includes the onset of aneurysms formation, atherosclerotic changes and progression until rupture. Nowadays, in vivo MPM of vessel function and morphology has been investigated only in rodents^44^ and miniaturized endoscopic systems have been employed for label-free colonoscopy of mice^45^ as well as for redox imaging of kidney ischemia–reperfusion model^46^. Latter research group used already a compact and flexible fiber-optic probe of about 2 mm diameter. Furthermore, clinically approved systems for label-free multiphoton analysis of the human skin already exist^47^. Taking into account further technical development towards clinical application, it might be possible to investigate aneurysm walls with endomicroscopes and possibly to predict the area of rupture in the future.

### Benefits

An extended set of special immunohistochemical stainings might be likewise able to visualize the tissue alterations occurring in aneurysm formation and progression. However, MPM has crucial advantages in comparison to conventional histological stainings: it does not require any fixation process and it is characterized by high specificity and high sensitivity^48^. In contrast, the quality of all conventional stainings is influenced by chemical fixation (e.g. methanol, acetone, formalin), the embedding process and the related chemicals (especially paraffin embedding-dehydration), antigen retrieval procedure (e.g. microwave) as well as the age and quality (i.e. how often they were used) of the different staining reagents/antibodies^49^. These steps may change the native morphology e.g. of elastin and collagen. Hence, quantification of fiber fragmentation, fiber length, disorganization and total volumetric density is difficult with conventional stainings but possible with label-free MPM that can be applied on fresh, unprocessed tissue^48^. Moreover, the assessment of different criteria of wall alterations requires the analysis of multiple histological stainings of individual sections. For example, Oil Red O stain can be used for investigation of lipids and cholesterol esters; Masson-Trichrome stain or EvG stain can address collagen and elastin. In contrast, MPM is able to detect simultaneously extra- and intracellular lipids, like lipid-laden cells, low-density lipoproteins such as cholesterol and cholesterol esters as well as collagen and elastin^13,25,28^.

## Limitations

Label-free MPM does not possess the ability of immunohistochemistry to address single epitopes with high specificity. At present, label-free MPM can visualize only a limited number of tissue components such as elastin, collagen and lipids. Furthermore, the required microscopy systems are rather expensive and commercial systems integrating CARS microscopy, for instance, are still of limited availability. Raman spectroscopy provides better chemical specificity for reference of MPM images, but, as it is a single-point measurement, it requires very long acquisition time for high-resolution spatial mapping. Other limitations of our study are the small specimen size as well as the aneurysm dome extraction. Only the aneurysm domes were removed after clipping, while the necks of the aneurysms remained and could not be addressed in this study. Furthermore, only one time point of aneurysm formation can be assessed for each patient. No progression studies are possible. Therefore, it is not possible to investigate the whole histopathogenesis of a cerebral aneurysm.

## Conclusion

In summary, we showed that label-free multiphoton microscopy is well suited to provide morphological and biochemical information that are crucial for understanding the pathological vessel wall changes of cerebral aneurysms on a macroscopic and microstructural level. Histopathological alterations of aneurysms and their atherosclerotic changes such as: wall thickening and thinning, intimal hyperplasia, fibromatous wall remodelling, loss or fragmentation of elastin fibers, intra-wall haemorrhage, calcification, cholesterol and lipid accumulation and deposits can be visualized. Hence, MPM is at least as informative as different conventional histological stainings. Mentionable, MPM is performed without any fixation, non-destructively, in real-time mode and simultaneously. MPM is able to image biological thick specimens ex vivo and in vivo. In the future, MPM might help to improve the understanding of the mechanisms of aneurysm formation and progression, which will support a better predictability of the area of rupture.

## Material and methods

### Samples

Cerebral human saccular aneurysm domes were obtained from surgery after clipping procedure (unruptured n = 5, ruptured n = 5). Written informed consent was obtained from all patients. The ethics committee at Dresden University Hospital of the TU Dresden (EK 890998) approved the study. Human samples of the cerebral arterial circle (Circulus arteriosus cerebri, circle of Willis) were extracted from clinical autopsy (anonymized patients). Samples from surgery were snap-frozen in liquid nitrogen and samples from autopsy were fixed in 4% formalin. Cryosections of 16 µm thickness were prepared either on SuperFrost Plus™ glass slides or on CaF_2_ slides for Raman spectroscopy.

### Label-free multiphoton imaging

The cryosections were rehydrated with PBS before imaging. The used multiphoton microscope system was described previously^10^. Briefly, the multiphoton microscope is formed by an upright microscope Axio Examiner Z.1 coupled to a laser scanning module LSM 7, a W Plan-Apochromat 20 × /1.0 objective and non-descanned detectors (all from Carl Zeiss Microscopy GmbH, Jena, Germany). Two erbium fiber lasers (Femto Fiber pro NIR and TNIR, Toptica Photonics AG, Gräfelfing, Germany) were used for excitation and have a pulse length of around 1 ps. The pump laser emitted at a wavelength of 781 nm and the Stokes laser for the CARS signal at 1005 nm. They were used to excite TPEF, SHG and CARS signal from the symmetric stretching vibration of methylene groups at 2850 cm^–1^. All nonlinear signals were excited and acquired simultaneously using the proper optical filtering. The CARS signal was collected using a band pass filter centered on 640 nm with bandwidth of 14 nm. The TPEF signal was obtained in reflection in the spectral range of 500–550 nm. The SHG signal was acquired in transmission with a band pass filter centered at 390 nm and bandwidth of 18 nm. The signals were combined as 8 bit RGB images (red channel: CARS; green channel: TPEF; blue channel: SHG). The acquisition of large areas was performed with a tiling procedure. Z-stacks were used in order to compensate uneven surfaces of samples, followed by maximum intensity projections to obtain the final images.

### Raman spectroscopy

The spectroscopy was performed with a RamanRxn spectrometer (Kaiser Optical Systems Inc., Ann Arbor, MI) coupled to a light microscope (DM2500 P, Leica Microsystems GmbH, Wetzlar, Germany). The system was described previously^50^. The laser power at the sample was 160 mW. Raman spectra were recorded by line measurements, using an integration time of 1 s and averaging of 20 accumulations. The spectral resolution was 4 cm^−1^ and the acquired spectral range was from 150 to 3250 cm^−1^. For processing of the spectroscopic data Matlab packages (MathWorks Inc., Natick, MA) were used. Afterwards baseline correction was performed to subtract the background fluorescence by applying the function “msbackadj.” The function is contained in the Bioinformatics Toolbox. Important bands were identified by visual inspection. The Raman spectra displayed are single representative spectra.

### Comparison of label-free multiphoton imaging and Raman spectroscopy

In this study, we used label-free MPM and Raman spectroscopy to investigate human cerebral aneurysms. Table 2 clarifies the differences between the two imaging approaches.

**Table 2:** Comparison of different imaging systems used for this study.

	Label-free MPM			Raman spectroscopy
	CARS	TPEF	SHG	
Excitation	Pulsed ps laser 781 nm and 1005 nm			Cw laser 785 nm
Lateral resolution	Subcellular, ~ 0.6 µm			Low, ~ 20 µm
Acquisition speed	Fast, imaging technology			Slow, seconds for one position
Biochemical sensitivity	Low, limited to specific biochemical groups and compounds			High, full biochemical Composition
Tissue preprocessing	Not required			Not required
Label-free	Yes			Yes
Potential in vivo technology	Yes			Yes

### Reference histopathology

Cryosections were stained either with hematoxylin & eosin (HE) or with Elastica van Gieson (EvG). HE was carried out according to the protocol supplied by the manufacturer (Merck, Darmstadt, Germany). EvG was conducted according to the staining kit’s protocol also supplied by the manufacturer (Carl Roth, Karlsruhe, Germany).

### Ethical approval

The study was approved by the ethics committee at Dresden University Hospital of the TU Dresden (EK 890998) and conducted in accordance to the Declaration of Helsinki.
